# Comparative Transcriptomic Analyses Revealed the Effects of Poly (I:C) on the Liver and Spleen of *Argyrosomus japonicus*

**DOI:** 10.3390/ijms23179801

**Published:** 2022-08-29

**Authors:** Anle Xu, Fei Han, Yuan Zhang, Tao Zhou, Tianxiang Gao

**Affiliations:** 1 Fisheries College, Zhejiang Ocean University, Zhoushan 316022, China; 2 Fisheries College, Ocean University of China, Qingdao 266003, China; 3 College of Ocean and Earth Sciences, Xiamen University, Xiamen 361102, China

**Keywords:** *Argyrosomus japonicus*, poly (I:C), comparative transcriptomic analyses, functional genes, immunity

## Abstract

Poly (I:C) can work as an immunostimulant and a viral vaccine; however, its functional mechanism in aquatic animals needs to be further investigated. In this study, comparative transcriptomic analyses were performed to investigate the effects of poly (I:C) on *Argyrosomus japonicus* at 12 h and 48 h postinjection. A total of 194 and 294 differentially expressed genes were obtained in the liver and spleen, respectively. At 12 h, poly (I:C) injection could significantly influence the function of the metabolism-related pathways and immune-related pathways in the liver through the upregulation of the genes *GST*, *LPIN*, *FOXO1*, *CYP24A1*, *ECM1,* and *SGK1*, and the downregulation of the genes *IL-1β*, *CXC19*, *TNFAIP3*, and *IRF1*. At 48 h, poly (I:C) could enhance the liver energy metabolism by upregulating the genes *TXNRD* and *ECM1*, while it also induced some injury in the cells with the downregulation of the genes *HBA* and *CYP24A1*. In the spleen, poly (I:C) could regulate the fish immunity and inflammatory response by upregulating the genes *DDIT4*, *C3*, *EFNA*, and *MNK*, and by downregulating the genes *ABCA1*, *SORT1*, *TNF*, *TLR2*, *IL8*, and *MHCII* at 12 h, and at 48 h, the poly (I:C) had a similar influence as that in the liver. Intersection analyses demonstrated that *CYP24A1* and *ECM1* were the main functional genes that contributed to the health of the liver. Ten and four genes participated in maintaining the health of the two tissues after 12 h and 48 h, respectively. In summary, our results provided a new insight into ploy (I:C) application in *A. japonicus*, and it also helped us to better understand the fish response mechanism to the viral vaccine injection.

## 1. Introduction

Poly (I:C) is a synthetic double-stranded RNA virus analogue, which is named polyriboinosinic polyribocytidylic acid. Research has shown that poly (I:C) could increase the killing effect of the natural killer cells and stimulate macrophages [1]. Moreover, it could protect aquatic animals, such as *Marsupenaeus japonicus* [2], *Crassostrea gigas* [3], *Larimichthys crocea* [4], *Sebastiscus marmoratus* [5], etc., against pathogenic microorganism infection. As a phenomenon, poly (I:C) can be an immunostimulant applied in aquaculture, but it is more than an immunostimulant because it can also function as a vaccine of the double-stranded RNA virus [6,7].

*Argyrosomus japonicus* is an important economic marine fish, and it has excellent economic traits, such as nutritious meat, low oxygen tolerance, and a high growth rate. It is widely cultured in South Africa, Australia, Japan, Korea, China, Pakistan and India [8]. At one time in China, *A. japonicus* was described as *Nibea japonicus* in some papers, but this was later corrected [9]. Therefore, reports on both *A. japonicus* and *N. japonicus* are on the same species. By now, as far as we know, most of the research on the fish is focused on the fields of nutrient and feed [10,11,12,13], environmental stress [14,15,16] and fishery resources [17,18,19], and research on the molecular-response mechanism induced by some beneficial substances (e.g., immunological adjuvants and vaccines) is rare [20]. 

In this study, poly (I:C) was intraperitoneally injected into *A. japonicus.* Then, the liver and spleen were dissected out of the body, and comparative transcriptomic analyses were performed for the two tissues. We aimed to investigate the effects of poly (I:C) on *A. japonicus*, including the differentially expressed genes and major functional pathways induced by poly (I:C) in the liver and spleen, which could provide new insight into the potential application of poly (I:C) in *A. japonicus* farming, and increase the understanding of the fish response mechanism to the viral vaccine injection.

## 2. Results 

### 2.1. Sequencing Data, Transcriptome Assembly and Gene Annotation

The sequencing results are summarized in Table S1. Twenty-four cDNA libraries were established for the study (twelve for the liver, and the others for the spleen). In the current study, from about 57.77 to 85.38 million clean reads for the liver, and from 54.78 to 77.80 clean reads for the spleen were obtained after the raw data were filtered. The Q20 and Q30 ratios in the liver ranged from 97.08% to 97.94%, and from 92.15% to 94%, respectively. Similar parameters were found in the spleen, from 96.65% to 97.79%, and from 91.26% to 93.69%, respectively, showing that the quality score of the sequencing data of both tissues was above 91%. The GC content of the liver and spleen varied from 46.76 to 51.04%, and from 46.76 to 48.36%, respectively, which displayed similar values to most eukaryotes. Matching the high-quality clean reads to the reference genome demonstrated that the mapping ratio of both tissues could reach over 93%.

A total of 23,947 unigenes were spliced out after removing the redundancy. The numbers of unigenes annotated in the eight databases are shown in Table 1. The highest annotation ratio was in the Nt database, where the value reached 96.88%, while the lowest was in the TFs database. The quality of the sequencing data met the demand for transcriptome analysis, and the results of the annotations with the eight databases were admissible. 

### 2.2. Analysis of Differentially Expressed Genes

**Figure 1:**
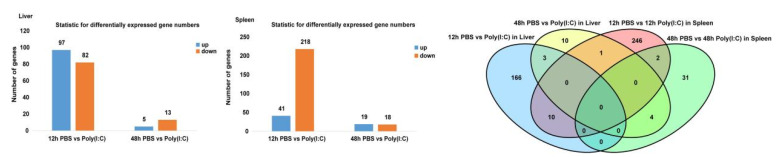
Numbers of Differentially Expressed Genes in Each Comparison, Followed by the Interaction Venn-diagram Analyses of these Comparisons.

Pairwise comparisons were performed between the control group (fish injected with PBS) and trial group (fish injected with poly (I:C)) at 12 h and 48 h postinjection. The numbers of DEGs obtained from the comparisons are presented in Figure 1. At 12 h after poly (I:C) injection, 179 DEGs (97 genes upregulated and 82 genes downregulated) were obtained from the liver, while from the spleen, the number of DEGs was 259, with 41genes upregulated and 218 genes downregulated. At 48 h postinjection of poly (I:C), 18 and 37 DEGs were detected in the liver and spleen, respectively. Interaction Venn-diagram analyses of the four comparisons were carried out, and the results demonstrated that, in the liver, a total of 194 DEGs were found due to poly (I:C) injection at 12 h and 48 h, and 3 genes were shared and joined forces with the different trends of the up- and downregulation at the two times (Table 2). Similarly, in the spleen, the numbers of genes were 294 and 2, respectively (Table 2). Ten DEGs were found to co-function in both tissues, with the same trend of up- and downregulation at 12 h, and four genes at 48 h (Table 3). 

### 2.3. GO-Enrichment Analysis of Differentially Expressed Genes

**Figure 2:**
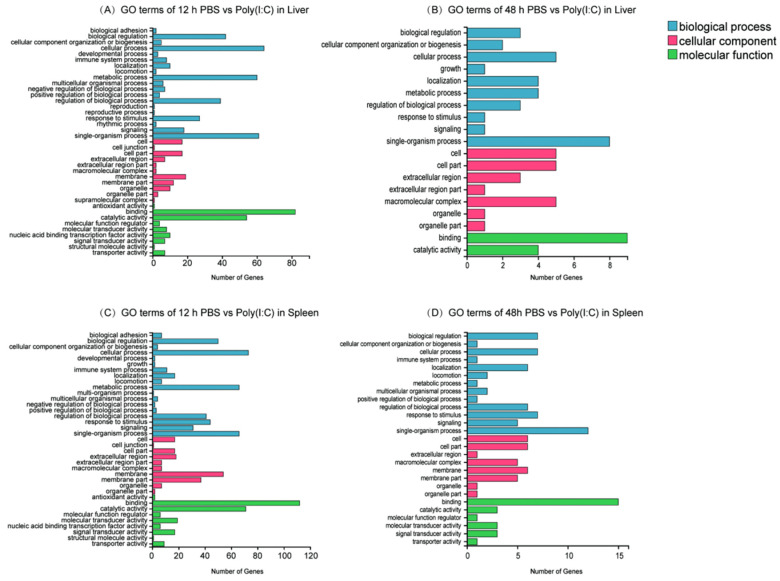
GO Enrichment of Differentially Expressed Genes in Both Tissues at Different Times: (**A**) Represents the Result of GO Enrichment in Comparative Case of 12 h PBS vs Poly (I:C) in the Liver; (**B**) Represents the Result of GO Enrichment in Comparative Case of 48 h PBS vs Poly (I:C) in the Liver; (**C**) Represents the Result of GO Enrichment in Comparative Case of 12 h PBS vs Poly (I:C) in the Spleen; (**D**) Represents the Result of GO Enrichment in Comparative Case of 48 h PBS vs Poly (I:C) in the Spleen.

In the liver, at 12 h of comparison, the 179 DEGs could be enriched into 835 GO terms, involving three categories, and the main enrichment terms are shown in Figure 2A,B. In the biological processes, the genes were mainly enriched in the cellular process, metabolic process and single-organism process. In the cellular components, the genes were mainly enriched in the membrane and cell parts. In the molecular functions, the genes were mostly enriched in binding and catalytic activities. Significant enrichment analysis demonstrated that these genes could be located in 21 GO terms (*q*-value < 0.05), such as the metabolic process, oxidoreductase activity, immune response and so on (Table S2). At 48 h of comparison, the 18 genes were enriched into 139 GO terms, and they were mainly enriched in the single-organism process, cell, cell part, macromolecular complex and binding (Figure 2C,D). The corresponding GO-enrichment analysis revealed that 22 terms were significantly enriched by these genes (Table S2).

In the spleen, 735 GO terms were enriched by the 259 DEGs at 12 h postinjection. The major terms that the genes were attributed to were cellular processing, membranes and binding, representing biological processes, and the cellular components and molecular functions. The genes could be significantly enriched into 67 terms (Table S2). At 48 h postinjection, the 37 DEGs could be enriched into 230 GO terms, and the major enriched terms were similar to those of the 12 h comparison. The number of the significant enriched terms was 12 (Table S2).

### 2.4. KEGG-Pathway-Enrichment Analysis of Differentially Expressed Genes

**Figure 3:**
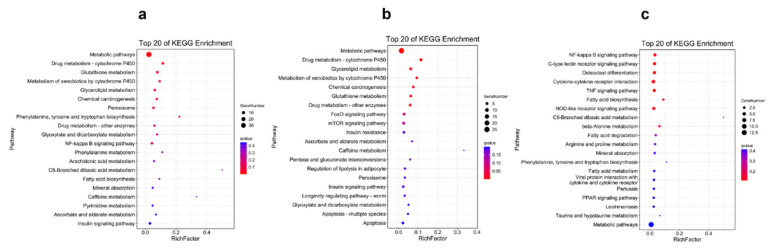
The Top 20 KEGG Pathways Enriched by the Differentially Expressed Genes in the Liver at 12 h Postinjection: (**a**) the Top 20 Enrichment Pathways among All Differentially Expressed Genes; (**b**) the Top 20 Enrichment Pathways among the Upregulated Differentially Expressed Genes; (**c**) the Top 20 Enrichment Pathways among the Downregulated Differentially Expressed Genes.

**Figure 4:**
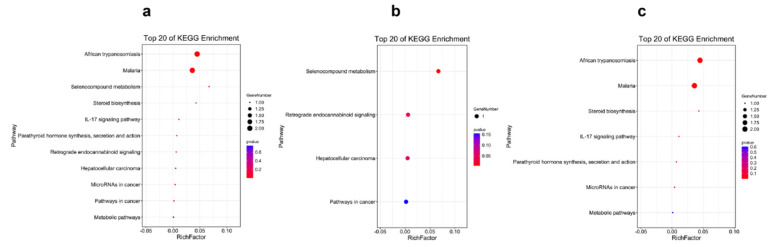
The Top 20 KEGG Pathways Enriched by the Differentially Expressed Genes in the Liver at 48 h Postinjection: (**a**) the Top 20 Enrichment Pathways among All Differentially Expressed Genes; (**b**) the Top 20 Enrichment Pathways among the Upregulated Differentially Expressed Genes; (**c**) the Top 20 Enrichment Pathways among the Downregulated Differentially Expressed Genes.

**Figure 5:**
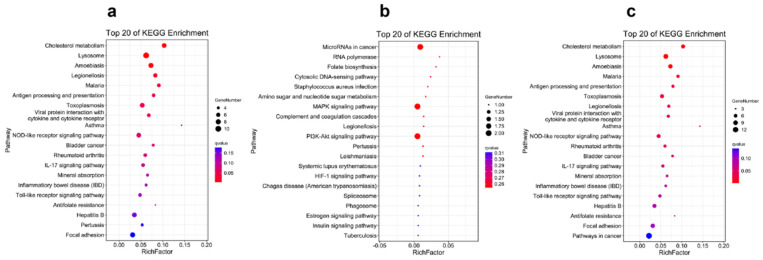
The Top 20 KEGG Pathways Enriched by the Differentially Expressed Genes in the Spleen at 12 h Postinjection: (**a**) the Top 20 Enrichment Pathways among All Differentially Expressed Genes; (**b**) the Top 20 Enrichment Pathways among the Upregulated Differentially Expressed Genes; (**c**) the Top 20 Enrichment Pathways among the Downregulated Differentially Expressed Genes.

**Figure 6:**
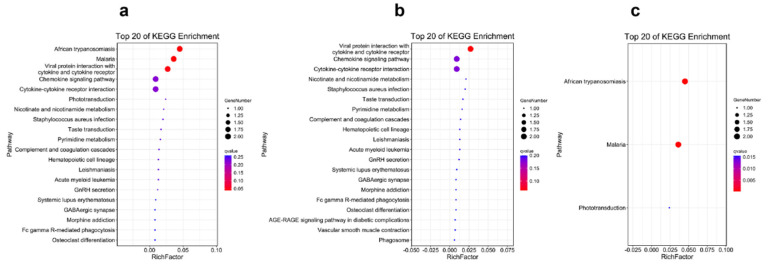
The Top 20 KEGG Pathways Enriched by the Differentially Expressed Genes in the Spleen at 48 h Postinjection: (**a**) the Top 20 Enrichment Pathways among All Differentially Expressed Genes; (**b**) the Top 20 Enrichment Pathways among the Upregulated Differentially Expressed Genes; (**c**) the Top 20 Enrichment Pathways among the Downregulated Differentially Expressed Genes.

The results of the KEGG pathway enrichment for the DEGs from different tissues are displayed in Figure 3, Figure 4, Figure 5 and Figure 6. For the comparison of 12 h postinjection, the DEGs obtained from the liver could be enriched into 202 pathways, and especially in the metabolism-related pathways, including drug metabolism (cytochrome P450), glutathione metabolism, glycerolipid metabolism, phenylalanine, tyrosine and tryptophan biosynthesis, fatty acid biosynthesis, etc., and immune-related pathways, such as peroxisomes and the NF-kappa B signaling pathway (Figure 3a). The upregulated genes could be enriched into 142 pathways, but they were mainly enriched into the metabolism-related pathways, and they could also be enriched into the immune-related pathways (FoxO signaling pathway and mTOR signaling pathway) (*p* < 0.05) (Figure 3b). The downregulated genes were mainly enriched into the immune-related pathways, such as the NF-kappa B signaling pathway, C-type lectin receptor signaling pathway, cytokine–cytokine-receptor interaction, the NOD-like receptor signaling pathway and so on (Figure 3c). At 48 h postinjection in the liver, it obtained 18 DEGs, which could be enriched into 11 pathways. The upregulated genes could only be enriched into four pathways, consisting of selenocompound metabolism, retrograde endocannabinoid signaling, hepatocellular carcinoma and pathways in cancer. The downregulated genes could be enriched into seven pathways, and the first four pathways that were significantly enriched were African trypanosomiasis, malaria, steroid biosynthesis and the IL-17 signaling pathway (*p* < 0.05) (Figure 4).

Figure 5 and Figure 6 display the pathway-enrichment-analysis results in the spleen at the two times. At 12 h, the 259 DEGs could be enriched into 219 pathways, which focused on the immune-related pathways, such as lysosome, antigen processing and presentation and the NOD-like receptor signaling pathway, and some disease-related pathways were also enriched, which provided evidence that the poly (I:C) might work as one vaccine. The pathway of cholesterol metabolism was also significantly enriched, which was probably related to the membrane or body-energy compensation against the stress. The upregulated genes could be enriched into 32 pathways, mainly in microRNAs in cancer, the MAPK signaling pathway and the PI3K-Akt signaling pathway (Figure 5b). The downregulated genes were enriched into 212 pathways, and mainly in the immune-related and disease-related pathways (Figure 5c). The results of the 48 h postinjection in the spleen revealed that the 37 DEGs could be enriched into 32 pathways that were related to disease and the inflammatory response (Figure 6a). The upregulated genes could be enriched into 29 pathways, and some genes functioned in the chemokine signaling pathway and cytokine–cytokine-receptor interaction (Figure 6b). The downregulated genes could only be enriched into three pathways, and they were African trypanosomiasis, malaria and phototransduction (Figure 6c).

**Figure 7:**
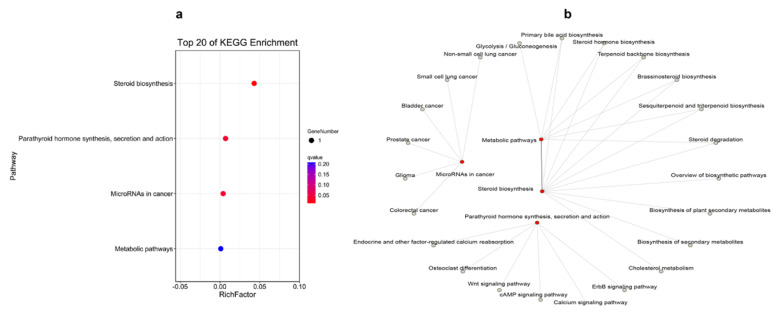
The Top 20 KEGG Pathways Enriched by the Differentially Expressed Genes of the Shared Genes at 12 h and 48 h Postinjection in the Liver, and the Network Graph among these Pathways: (**a**) the Pathway Enrichment of the Shared Differentially Expressed Genes; (**b**) the Network Graph among these Pathways.

**Figure 8:**
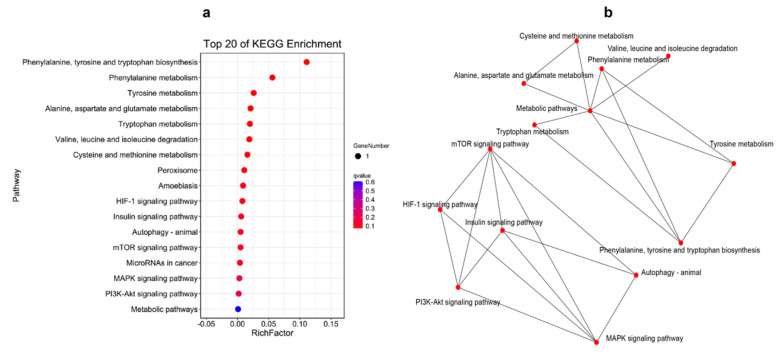
The Top 20 KEGG Pathways Enriched by the Differentially Expressed Genes of the Shared Genes Between the Liver and Spleen at 12 h Postinjection, and the Network Graph among these Pathways: (**a**) the Pathway Enrichment of the Shared Differentially Expressed Genes; (**b**) the Network Graph among these Pathways.

**Figure 9:**
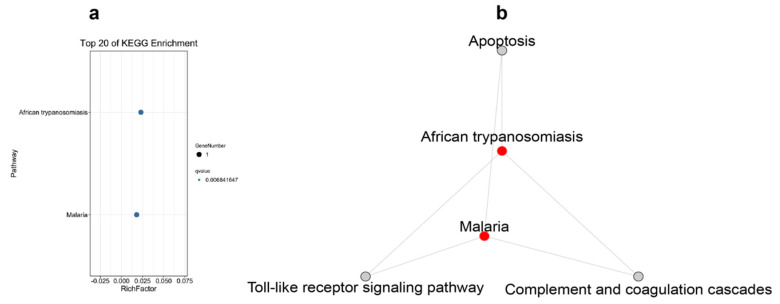
The Top 20 KEGG Pathways Enriched by the Differentially Expressed Genes of the Shared Genes Between the Liver and Spleen at 48 h Postinjection, and the Network Graph among these Pathways: (**a**) the Pathway Enrichment of the Shared Differentially Expressed Genes; (**b**) the Network Graph among these Pathways.

KEGG-pathway-enrichment analyses were also performed for the shared DEGs in different cases. Three genes were co-functioning in the liver at different times, and they could be enriched into four pathways, which could regulate the interactive pathways (shown in Figure 7). Two genes were found to have worked in the spleen at 12 h and 48 h, but without pathway enrichment. There were ten shared genes engaged in responding to poly (I:C) injection in both the liver and spleen at 12 h. They were enriched into 17 pathways, which were mostly focused on amino acid metabolism and some oxidative-stress response, which had separated interactions (Figure 8). Figure 9 demonstrates that the shared genes in both tissues at 48 h could be enriched into two pathways, which could regulate interactively with apoptosis, the Toll-like receptor signaling pathway and complement and coagulation cascades.

### 2.5. Differentially-Expressed-Gene Verification by qRT-PCR

**Figure 10:**
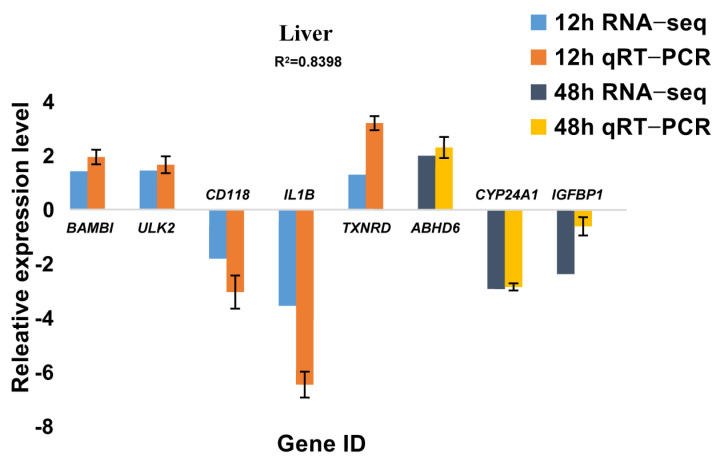
Results of qRT−PCR for Some Differentially Expressed Genes in the Liver.

**Figure 11:**
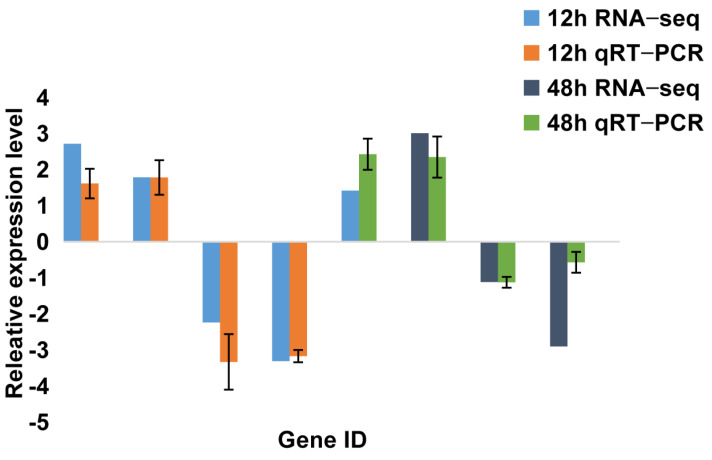
Results of qRT−PCR for Some Differentially Expressed Genes in the Spleen.

In order to verify the accuracy of the sequencing results and identify the reliability of the analysis, sixteen DEGs from the four comparisons (four genes from each comparison) were selected for qRT-PCR. The relative gene expressions were counted using the −ΔΔCt method, and the results were then compared with the RNA-seq results. Our current results showed that all the relative-gene-expression levels had a similar trend to the RNA-seq (Figure 10 and Figure 11), which suggested that the results provided by the RNA-seq were generally reliable.

## 3. Discussion

Poly (I:C) could function as an immunostimulant and as a vaccine of double-stranded RNA virus, improving some fish immunity [2,3,4,5]. However, the effects of poly (I:C) on *A. japonicus,* as well as the molecule-responsive mechanism of the virus infection, which commonly occurs in *A. japonicus*, have not been reported. It is meaningful to investigate the potential application of poly (I:C), and to enrich the knowledge about the molecular mechanism induced by the double-stranded RNA virus infection via the analogue in *A. japonicus*, and to thus propose some measures for the sake of the healthy aquaculture of the fish. In the current study, both the liver and spleen were selected to reach our goal, and comparative transcriptome analyses were performed. The results of the qRT-PCR demonstrated that the transcriptome sequencing and analysis were reliable. 

In many stress trials, the 12 h and 48 h post-treatment are two important observation times [5,21,22,23], and so we used them in our current study. At 12 h postinjection of poly (I:C) in the liver, the 179 DEGs could be enriched into 202 pathways, and especially the metabolism-related pathways. *GST* plays important roles in detoxification, and its regulated enzyme product also can help to eliminate radicals [24,25]. In our study, poly (I:C) injection significantly increased the fish *GST* expression, compared with the control group, indicating that *GST* might sensitively react against xenobiotics and keep the host healthy [26]. The glycerolipid metabolism pathway was significantly enriched, and the *LPIN* gene was upregulated, which is a similar result to the research on *Sparus aurata* induced by metformin [27], revealing that fish might have metabolic compensation when some xenobiotics invade the body; the phenomenon has also been presented in previous research [28]. The immune-related pathways, such as the FoxO signaling pathway and mTOR signaling pathway, were enriched, and the genes *FOXO1* and *SGK1* were upregulated, which affected the cell survival and mitosis. The GO-enrichment analysis showed that the DEGs could be significantly enriched in biological processes related to the immune response (GO:0006955) and the immune-system process (GO:0002376), as well as in molecular functions related to oxidative stress (GO:0016705) and inflammation (GO:0005125, GO:0005125). Both the pathways and GO enrichment related to immunity elucidated that poly (I:C) might regulate *FOXO1* and *SGK1* genes, as well as stimulate some enzymes to protect the liver cells and maintain its normal function [29,30]. The downregulated genes were mostly enriched in the immune-related pathways, and the genes included *IL-1β*, *CXC19*, *TNFAIP3* and *IRF1*. Research has uncovered that the upregulation of these genes is associated with inflammation, which is bad for the body [31,32,33]. In our study, these genes were downregulated, which suggests that poly (I:C) might weaken the inflammation of the liver. This finding matches other research on poly (I:C) stress in aquatic animals [5,29], and it also coincides with the phenomenon of the negative correlation between the genes *IL-1β* and *BAMBI*, *ULK2*, which could active autophagy and inhibit inflammation [34,35]. At 48 h postinjection, the 18 DEGs could be enriched into the disease-related pathways and steroid biosynthesis, and the major functional genes included *TXNRD* (upregulation), and *HBA* and *CYP24A1* (downregulation). The upregulation of *TXNRD* is associated with the high activity of mitochondria [36]; however, it might produce a large amount of reactive oxygen species [37], which would injure the cell. *HBA* and *CYP24A1* play important roles in protecting cells in fish [38,39], and their downregulation is probably related to the damage of cells. This speculation could also be found in some of the evidence provided by the GO enrichment, which showed that the DEGs were mainly enriched in the cellular components. As a consequence, we conjectured that, at 48 h postinjection, poly (I:C) could enhance the liver energy metabolism by increasing the activity of mitochondria, but, in return, it could cause some injury to the cells.

In aquatic animals, the spleen has been reported as a major organ that participates in body immunity [40]. Hence, in our study, we selected it to perform an analysis of poly (I:C) injection. The results displayed that poly (I:C) stress produced 259 and 37 DEGs at 12 h and 48 h, respectively. At 12 h, the upregulated genes could be enriched in the pathways of microRNAs in cancer, the MAPK signaling pathway, the PI3K–Akt signaling pathway and complement and coagulation cascades, and the genes included *DDIT4*, *C3*, *EFNA*, *MNK*, etc. It was proven that the upregulation of *DDIT4* could promote cell proliferation and mediate the anticancer effect. Both *DDIT4* and *C3* could regulate the *Epinephelus fuscoguttatus* × *Epinephelus lanceolatus* skin color, which contributed to the better living of fish in the environment [41]. The *MNK* upregulation could affect the TL-17 signaling pathway and then increase fish immunity [42]. Based on these results, we inferred that poly (I:C) could enhance *A. japonicus* immunity and adaptation to the environment; similar results on the effects of poly (I:C) were also found in other aquatic animals [1,3,5]. The downregulated genes could be significantly enriched in the pathways of cholesterol metabolism, lysosomes, antigen processing and presentation, the NOD-like receptor signaling pathway and some disease-related pathways (*p* < 0.05). Poly (I:C) is a synthetic double-stranded RNA virus analogue, and it probably causes a series of responses, such as virus invasion after injection. Hence, the genes *ABCA1* and *SORT1* (functioning in cholesterol metabolism), which could affect lipid abnormalities and hematopoietic function [43,44], were downregulated. The genes *TNF*, *TLR2*, *IL8* and *MHCII* in these pathways were also downregulated. They are associated with the body function of the natural cell-killing ability and T-cell immunity, and their downregulation demonstrated that poly (I:C) would regulate these genes, and then enhance the natural cell-killing ability, affect the T-cell immunity and decrease apoptosis induced by endogenesis [1,45,46,47]. At 48 h, poly (I:C) injection might, on the one hand, enhance the spleen energy metabolism, while, on the other hand, induce some injury, such as virus infection, which is a finding that could be supported by the results that found that the disease-related pathways were significantly enriched, the damaged presentation gene *TXNRD* was upregulated [36] and *HBA* was downregulated [38].

The shared DEGs in the liver at 12 h and 48 h postinjection of poly (I:C) could be enriched in: steroid biosynthesis; parathyroid hormone synthesis, secretion and action; microRNAs in cancer; metabolic pathways. As can be seen from the associated network of the pathways (Figure 7b), it focused on metabolism, and this could be due to the fact that the liver was the main metabolic organ. The gene *CYP24A1* might affect the stability of the cell membrane [39], while the *ECM1* gene keeps the liver healthy [48], and they both maintained the liver functioning normally when injected with poly (I:C). No pathway was enriched in the spleen, according to the shared gene enrichment analysis result, showing that the fish could have an unconnected regulatory-mechanism response to poly (I:C) injection at different times in this tissue. At 12 h in the two tissues, ten shared DEGs were found, and they were mainly enriched in the metabolism-related pathway; meanwhile, the interactive relationship among these pathways provided further evidence that poly (I:C) could affect the tissues’ metabolism and immunity. The network graph, supported by the shared DEGs in both tissues at 48 h, also proved that the poly (I:C) could serve as a vaccine applied to *A. japonicus*, and it might develop the fish’s disease-resistance ability by influencing the pathways of apoptosis, the Toll-like receptor signaling pathway and complement and coagulation cascades. 

## 4. Materials and Methods

### 4.1. Ethic Statement

This study was conducted following the National Research Council’s guide for the care and use of laboratory animals, and it was approved by the Animal Ethical Committee of Zhejiang Ocean University (Zhejiang, China).

### 4.2. Fish Handling and Trial Design

*Argyrosomus japonicus* were captured offshore of Shengsi Island, Zhoushan city, Zhejiang Province, China. The fish were acclimated under the following environmental conditions: a temperature of 24 ± 1 °C, and a salinity of 24 ppt, which were suitable for the living fish [14], until no dead fish occurred daily.

Sixty healthy individuals with a similar size (average weight: 50 ± 5 g) were randomly divided into two groups, at 30 fish per group. Each fish of the control group was intraperitoneally injected with 200 μL PBS reagent, while the treatment group was injected with 200 μL of 2 mg/mL poly (I:C) (the reagents were both supported by Beyotime Biotechnology, Shanghai, China). The sampling time referred to the previous research [5,21]. Briefly, each group contained three biological replicates, with a total of 9 fish (3 fish as a sample, *n* = 3), and the fish were dissected to collect the liver and spleen samples at 12 h and 48 h postinjection. The liver or spleen from each fish was quickly put into a single frozen pipe and was snap-frozen in the liquid nitrogen, and finally transported to store at −80 °C until use.

### 4.3. RNA Extraction, Library Construction, Sequencing and Read Mapping with Reference Genome

The liver and spleen RNA of each fish were separately extracted, and the RNA of each biological replicate was composed of an equal mass mixing of the RNA from the three fish. All RNA extractions were performed using TRlzol reagent (Vazyme, Nanjing, China), and then the integrity and purity were identified via 1% (*w/v*) agarose gel, a Nanodrop 1000 spectrophotometer (ThermoFisher Scientific, Waltham, MA, USA) and Agilent 2100 (Agilent, Agilent Technologies, Inc, Carlsbad, CA, United States). RNA samples with high quality (A260/A280 > 1.8; A260/A230 > 1.8 (Table 4)) were picked for the library construction and high-throughput sequencing.

After enrichment with oligo-dT beads, the purified mRNA of each sample was treated with the NEBNext^®^ UltraTM RNA Library Prep Kit for Illumina^®^ (NEB, Ipswich, MA, USA), according to the manufacturer’s instructions, and we subsequently completed the first-strand-cDNA and second-strand-cDNA synthesis. The double-stranded cDNA was purified, end repaired and added to the poly A tail, and next, the sequencing joint was attached. Finally, the product was purified again, and the library quality was evaluated by an Agilent 2100 system first, and then, if applicable, the 24 cDNA libraries were sequenced on an Illumina HiSeq 2000; the paired-end read was 125 bp/150 bp (Novogene, Beijing, China). All raw reads produced in the research were submitted to the National Centre for Biotechnology Information (NCBI) Sequence Read Archive database (SRA, NCBI, Bethesda Maryland, USA) with the accession number: PRJNA838245.

### 4.4. Assembly of Transcriptome and Functional Annotation

The sequencing data were filtered to remove reads with adaptors, unknown nucleotides of an “N” base > 10% and low-quality reads (a read with a quality score < 15, and the percentage of bases in the read exceeds 50%), and then high-quality clean reads were obtained. The Q20 (percentage of the Phred quality score > 20) and Q30 (percentage of Phred quality score > 30) ratios were calculated for the sequencing quality analysis.

The high-quality clean reads were compared with the reference genome of *A. japonicus* (GCA_015710095.1) by the short-read-alignment tool of Bowtie 2 (version 2.2.5) (SourceForge, San Diego, USA), and then the expression levels of the genes were calculated via RSEM software, and they were described with fragments per kilobase of the exon model per million mapped fragments (FPKM) [49]. The gene functions were annotated according to the protein databases below: Nr (nonredundant-protein-sequence database), Nt (nucleotide-sequence database), TFs (animal-transcription-factor database), Swiss-Prot (a manually annotated and reviewed protein-sequence database), Pfam (protein-family database), KOG/COG (http://www.ncbi.nlm.nih.gov/COG/, 22 April 2020), GO (http://www.geneontology.org/, 23 March 2022) and KEGG (http://www.genome.jp/kegg/, 23 March 2022) by blastx, with an E-value < 10^−5^. Blast2GO was used to finish the gene ontology (GO), and the pathway predictions were based on the results of the gene KEGG annotation.

### 4.5. Identification of Differentially Expressed Genes (DEGs) and Enrichment Analysis

The differential expression analysis between the groups in the two tissues (liver: 12 h PBS vs. poly (I:C), and 48 h PBS vs. poly (I:C); spleen: 12 h PBS vs. poly (I:C), and 48 h PBS vs. poly (I:C)) were performed by the DEG method [50]. The significant *p*-value was corrected for the false-discovery rate, and the adjusted *p*-value (Padj), which is named the *q*-value, is the FDR-corrected *p*-value. The significant-differential-expression level was set as a *q*-value < 0.05, and genes were considered to be significantly differentially expressed when the log_2_ (fold change) ≥ 1.

The GO enrichment of DEGs was analyzed with GOseq, which mapped the DEGs to the GO database, and divided their affiliate terms into three categories: biological processes, cellular components and molecular functions. The pathway-enrichment analyses of the DEGs referred to the method of the KEGG annotation of DEGs [51]. For the two enrichment analyses, a *q*-value < 0.05 demonstrated that the DEGs were significantly enriched in the GO terms and KEGG pathway, and the smaller the value, the more obvious the enrichment.

### 4.6. Transcriptome Verification with qRT-PCR

To verify the veracity and reliability of the transcriptomic results, quantitative real-time PCR was performed. A total of sixteen DEGs (four genes for each comparison) were screened out for qRT-PCR analysis using a StepOne Plus Real-Time PCR system (ABI 7500, ABI Company, Los Angeles, CA, USA), and the operational process obeyed the instruction of TaKaRa TB Green Premix Ex Taq (Til RAase Plus, RR420A) [8]. In order to decrease the experimental error, the verified DEGs were randomly selected, but they still needed to meet the following requirements: an FPKM of the gene > 1, and the gene should function in at least one pathway. All primers (sequences are shown in Table S3) used for the qRT-PCR were designed via primer 5.0 software, and *β-actin* was set as the reference gene. A melting-curve analysis was performed to confirm the primer specificity, and three replications for each gene were performed to obtain the Ct value. The relative expression levels of the target genes were calculated according to the −ΔΔCt method [52]. Statistical Package for Social Science, release 22.0 (SPSS 22.0, IBM Company, Chicago, IL, USA), was used to finish the statistical analysis.

## 5. Conclusions

Overall, a total of 194 and 294 DEGs were identified in the liver and spleen, respectively, in *A. japonicus* after poly (I:C) injection. In the liver, at 12 h, the poly (I:C) injection could significantly influence the metabolism-related pathway and immune-related pathway, and the main genes included *GST*, *LPIN*, *FOXO1*, *SGK1*, *BAMBI*, *IL-1β*, *CXC19*, *TNFAIP3* and *IRF1*. At 48 h, poly (I:C) could enhance the liver energy metabolism by upregulating the gene *TXNRD*, and it also induced some injury in the cells with the downregulation of the genes *HBA* and *CYP24A1*. In the spleen, at 12 h, poly (I:C) could regulate the fish immunity by upregulating the genes *DDIT4*, *C3*, *EFNA* and *MNK*, and by downregulating the genes *ABCA1*, *SORT1*, *TNF*, *TLR2*, *IL8* and *MHCII*. At 48 h, poly (I:C) could affect the fish health by upregulating the gene *TXNRD* and downregulating the gene *HBA*. In summary, our results revealed the fish response mechanism to poly (I:C), and it provided evidence and data that support the application of the substance in the healthy culturing of the fish.

## Figures and Tables

**Table 1:** Results of Unigene Annotation with the Eight Databases.

Database	Number of Unigenes	Ratio (%)
KEGG	14,233	59.44
GO	15,857	66.22
Nr	22,879	95.54
Nt	23,199	96.88
Swiss-Prot	22,831	95.34
Pfam	20,173	84.24
Kog	18,403	76.85
TFs	3,579	14.95
Total	23,947	100

**Table 2:** The Shared Differentially Expressed Gene Information of Tissues at Different Times.

Gene ID	Symbol	12 h		48 h	
		Up	Down	Up	Down
**in Liver**					
gene_Nib0013800.1	*CYP24A1*	+			+
gene_Nib0077620.1	*ECM1*	+		+	
gene_Nib0141020.1	*H1_5*	+			+
**in Spleen**					
gene_Nib0070130.1	Unannonated		+		+
gene_Nib0217840.1	Unannonated		+	+	

Note: The “+” demonstrates that the genes existed in this column, and the same below.

**Table 3:** The Shared Differentially Expressed Gene Information of Different Tissues at the Same Time.

Gene ID	Symbol	Liver		Spleen	
		Up	Down	Up	Down
**12h Liver ∩ Spleen**					
gene_Nib0213270.1	*CD50*		+		+
gene_Nib0109170.1	*IL4I1*		+		+
gene_Nib0066250.1	*DDIT4*	+		+	
gene_Nib0081460.1	*IL17_R_N*		+		+
gene_Nib0045660.1	*NR1D2*	+		+	
gene_Nib0052890.1	*IEX-1*		+		+
gene_Nib0001070.1	*MFHAS1*		+		+
gene_Nib0088750.1	*MNK*	+		+	
gene_Nib0002380.1	*PDZ*		+		+
gene_Nib0021160.1	*PRDX1*		+		+
**48h Liver ∩ Spleen**					
gene_Nib0170470.1	*HBA*		+		+
gene_Nib0170480.1	*HBE*		+		+
gene_Nib0170440.1	*HBA2*		+		+
gene_Nib0228720.1	*DMBT1*		+		+

Note: The “+” demonstrates that the genes existed in this column, and the same below.

**Table 4:** The Quality-inspection Results of All RNA Samples.

Sample Name	A260/A280	A260/A230	RIN
12h PBS-1 Liver	2.05	2.41	8.3
12h PBS-2 Liver	2.02	2.44	9.7
12h PBS-3 Liver	1.98	2.69	9.7
12h Poly-1 Liver	1.99	2.18	7.9
12h Poly-2 Liver	2.09	2.19	8.2
12h Poly-3 Liver	1.99	2.44	7.4
12h PBS-1 Spleen	2.03	2.8	9.4
12h PBS-2 Spleen	1.95	2.61	9.6
12h PBS-3 Spleen	1.95	4.56	9.3
12h Poly-1 Spleen	2.08	3.57	9.5
12h Poly-2 Spleen	2.05	3.22	9
12h Poly-3 Spleen	1.94	5.42	9.4
48h PBS-1 Liver	2.08	2.45	9.5
48h PBS-2 Liver	2.04	2.38	9.7
48h PBS-3 Liver	1.51	1.36	8.2
48h Poly-1 Liver	2.04	2.42	9.2
48h Poly-2 Liver	2.02	2.48	8.8
48h Poly-3 Liver	1.92	2.04	8.1
48h PBS-1 Spleen	1.92	3.2	10
48h PBS-2 Spleen	1.92	2.5	10
48h PBS-3 Spleen	2.1	3.81	8.9
48h Poly-1 Spleen	2.14	1.66	9.6
48h Poly-2 Spleen	2.01	3.42	9.7
48h Poly-3 Spleen	2.11	3.28	9.4

## Data Availability

All data and figures in the paper are available upon request.

## Supplementary Materials

The following supporting information can be downloaded at: https://www.mdpi.com/article/10.3390/ijms23179801/s1.
