# Supplementary material for: Comparative Transcriptomic Analyses Revealed the Effects of Poly (I:C) on the Liver and Spleen of Argyrosomus japonicus
Source: Int J Mol Sci. 2022 Aug 29;23(17):9801. doi: 10.3390/ijms23179801 (PMC9455969; doi:10.3390/ijms23179801)
Supplement: Supplementary file 1 [file ijms-23-09801-s001.zip › ijms-1863479-supplementary.pdf]

Table S1. Summary of the Transcriptome Sequencing and Mapping for *Argyrosomus japonicus.*

Comparison	Sample	Clean reads	Q20 (%)	Q30(%)	Clean reads Ratio(%)	GC(%)	Total mapping (%)
**12h PBS vs Poly(I:C) in liver**	PBS-1	85294154	97.81	93.71	99.33	49.73	96.67
	PBS-2	66547562	97.08	92.19	98.92	49.67	96.44
	PBS-3	63019164	97.11	92.22	99.12	50.15	96.74
	Poly (I:C)-1	78321342	97.49	92.94	99.23	49.97	96.57
	Poly (I:C)-2	85378004	97.18	92.44	98.82	50.40	96.47
	Poly (I:C)-3	63920620	97.34	92.62	99.12	46.76	95.16
**48h PBS vs Poly(I:C) in liver**	PBS-1	56772214	97.65	93.32	99.38	49.91	97.01
	PBS-2	80087902	97.08	92.15	99.02	49.91	96.61
	PBS-3	69516702	97.14	92.29	99.06	50.49	96.79
	Poly (I:C)-1	67711596	97.21	92.58	98.93	51.04	96.35
	Poly (I:C)-2	82903942	97.94	94.00	99.38	50.27	97.00
	Poly (I:C)-3	72540602	97.77	93.59	99.37	50.68	97.28
**12h PBS vs Poly(I:C) in spleen**	PBS-1	77804380	97.39	92.72	99.20	47.96	95.66
	PBS-2	60899134	97.46	92.85	99.32	48.05	95.71
	PBS-3	73946648	97.01	92.12	98.75	48.03	95.25
	Poly (I:C)-1	58408936	96.65	91.33	98.76	46.76	93.84
	Poly (I:C)-2	54779558	96.67	91.33	98.84	47.44	95.26
	Poly (I:C)-3	73061046	97.79	93.69	99.3	47.46	95.2
**48h PBS vs Poly(I:C) in spleen**	PBS-1	64535184	97.45	92.82	99.25	48.24	95.24
	PBS-2	65469666	97.43	92.82	99.21	48.01	95.45
	PBS-3	63655656	96.63	91.26	98.69	48.05	95.25
	Poly (I:C)-1	68098146	97.39	92.74	99.2	48.36	95.92
	Poly (I:C)-2	59624480	97.13	92.28	98.97	48.14	95.35
	Poly (I:C)-3	72596074	97.45	92.87	99.23	48.30	95.51

Table S2. Significantly Enriched Terms of the Differentially Expressed Gene in the Two Tissues at Different Times.

GO ID	Term	Ontology	q-value
	**12h PBS vs Poly(I:C) in liver**		
GO:0044699	single-organism process	P	2.58E-05
GO:0008152	metabolic process	P	5.34E-05
GO:0055114	oxidation-reduction process	P	0.000277044
GO:0044710	single-organism metabolic process	P	0.000443022
GO:0009987	cellular process	P	0.002002031
GO:0006955	immune response	P	0.008382679
GO:0002376	immune system process	P	0.01919847
GO:0065007	biological regulation	P	0.02063843
GO:0050789	regulation of biological process	P	0.04660546

GO:0050794	regulation of cellular process	P	0.04660546
GO:0032991	macromolecular complex	C	0.04660546
GO:0005488	binding	F	5.34E-05
GO:0016491	oxidoreductase activity	F	5.34E-05
GO:0003824	catalytic activity	F	0.000443022
GO:0005506	iron ion binding	F	0.008772117
GO:0004974	leukotriene receptor activity	F	0.01770242
GO:0016705	oxidoreductase activity, acting on paired donors, with incorporation or reduction of molecular oxygen	F	0.01856052
GO:0004731	purine-nucleoside phosphorylase activity	F	0.04660546
GO:0005125	cytokine activity	F	0.04660546
GO:0005126	cytokine receptor binding	F	0.04660546
GO:0020037	heme binding	F	0.04660546
	**48h PBS vs Poly(I:C) in liver**		
GO:0015669	gas transport	P	1.57E-08
GO:0015671	oxygen transport	P	1.57E-08
GO:0044765	single-organism transport	P	0.002703021
GO:1902578	single-organism localization	P	0.002868989
GO:0044699	single-organism process	P	0.01036064
GO:0005833	hemoglobin complex	C	1.57E-08
GO:0044445	cytosolic part	C	3.08E-08
GO:0005829	cytosol	C	1.48E-07
GO:0032991	macromolecular complex	C	0.00946551
GO:0044444	cytoplasmic part	C	0.00946551
GO:0043234	protein complex	C	0.01351179
GO:0005737	cytoplasm	C	0.019133
GO:0005576	extracellular region	C	0.02752409
GO:0019825	oxygen binding	F	1.97E-08
GO:0020037	heme binding	F	1.01E-07
GO:0046906	tetrapyrrole binding	F	1.02E-07
GO:0005506	iron ion binding	F	3.13E-07
GO:0046914	transition metal ion binding	F	0.002703021
GO:0043169	cation binding	F	0.004505831
GO:0046872	metal ion binding	F	0.004505831
GO:0043167	ion binding	F	0.01528579
GO:0097159	organic cyclic compound binding	F	0.02302566
GO:1901363	heterocyclic compound binding	F	0.02302566
GO:0005488	binding	F	0.04125839
	**12h PBS vs Poly(I:C) in spleen**		
GO:0002376	immune system process	P	0.002074306
GO:0055114	oxidation-reduction process	P	0.002074306
GO:0006955	immune response	P	0.002116746
GO:0006879	cellular iron ion homeostasis	P	0.002319678
GO:0046916	cellular transition metal ion homeostasis	P	0.00258743
GO:0055072	iron ion homeostasis	P	0.002790278
GO:0055076	transition metal ion homeostasis	P	0.003944179
GO:0044699	single-organism process	P	0.004054567
GO:0050896	response to stimulus	P	0.005516332
GO:0008152	metabolic process	P	0.007092808
GO:0050801	ion homeostasis	P	0.007092808
GO:0055080	cation homeostasis	P	0.007092808
GO:0098771	inorganic ion homeostasis	P	0.007092808
GO:0040011	locomotion	P	0.008479991
GO:0006873	cellular ion homeostasis	P	0.009039879
GO:0006875	cellular metal ion homeostasis	P	0.009039879
GO:0018101	protein citrullination	P	0.009039879
GO:0030003	cellular cation homeostasis	P	0.009039879
GO:0018195	peptidyl-arginine modification	P	0.01375888
GO:0055065	metal ion homeostasis	P	0.01375888
GO:0048878	chemical homeostasis	P	0.01759136
GO:0045995	regulation of embryonic development	P	0.02047182
GO:0006139	nucleobase-containing compound metabolic process	P	0.02431464

GO:0055082	cellular chemical homeostasis	P	0.0257757
GO:0044249	cellular biosynthetic process	P	0.03439493
GO:0065007	biological regulation	P	0.03439493
GO:0009987	cellular process	P	0.03481549
GO:0016477	cell migration	P	0.03940018
GO:0005576	extracellular region	C	0.002074306
GO:0043227	membrane-bounded organelle	C	0.00258743
GO:0043226	organelle	C	0.00314499
GO:0043231	intracellular membrane-bounded organelle	C	0.00314499
GO:0043229	intracellular organelle	C	0.003357993
GO:0016020	membrane	C	0.004610778
GO:0005622	intracellular	C	0.007092808
GO:0005634	nucleus	C	0.009307574
GO:0005623	cell	C	0.009434703
GO:0044464	cell part	C	0.009434703
GO:0044424	intracellular part	C	0.01038762
GO:0044422	organelle part	C	0.01468469
GO:0044446	intracellular organelle part	C	0.01627851
GO:0016021	integral component of membrane	C	0.02527139
GO:0031224	intrinsic component of membrane	C	0.0257757
GO:0044444	cytoplasmic part	C	0.02592009
GO:0005488	binding	F	2.03E-06
GO:0003824	catalytic activity	F	0.000438117
GO:0005125	cytokine activity	F	0.000812729
GO:0008009	chemokine activity	F	0.001182203
GO:0042379	chemokine receptor binding	F	0.001182203
GO:0016491	oxidoreductase activity	F	0.002074306
GO:0001664	G-protein coupled receptor binding	F	0.002790278
GO:0005126	cytokine receptor binding	F	0.00314499
GO:0046872	metal ion binding	F	0.008479991
GO:0043169	cation binding	F	0.008873332
GO:0004668	protein-arginine deiminase activity	F	0.009039879
GO:0016787	hydrolase activity	F	0.009039879
GO:0005515	protein binding	F	0.01024274
GO:0043167	ion binding	F	0.01255766
GO:0003677	DNA binding	F	0.01375888
GO:0005102	receptor binding	F	0.01375888
GO:0016494	C-X-C chemokine receptor activity	F	0.02047182
GO:0046914	transition metal ion binding	F	0.02308873
GO:0016813	hydrolase activity, acting on carbon-nitrogen (but not peptide) bonds, in linear amidines	F	0.02673186
GO:0005507	copper ion binding	F	0.03439493
GO:0004175	endopeptidase activity	F	0.03685261
GO:0005506	iron ion binding	F	0.0390501
GO:0004222	metalloendopeptidase activity	F	0.040739
	**48h PBS vs Poly(I:C) in spleen**		
GO:0015669	gas transport	P	6.56E-07
GO:0015671	oxygen transport	P	6.56E-07
GO:0005833	hemoglobin complex	C	6.56E-07
GO:0044445	cytosolic part	C	1.28E-06
GO:0005829	cytosol	C	8.13E-06
GO:0019825	oxygen binding	F	8.25E-07
GO:0020037	heme binding	F	0.0003801
GO:0046906	tetrapyrrole binding	F	0.0003801
GO:0005506	iron ion binding	F	0.000981324
GO:0001637	G-protein coupled chemoattractant receptor activity	F	0.01015893
GO:0004950	chemokine receptor activity	F	0.01015893
GO:0004896	cytokine receptor activity	F	0.0177785

Note: P represents biological, C represents cellular component and F represents molecular function.

Table S3. Primer Sequences Used for qRT-PCR.

Gene ID	Symbol		Primer
Reference gene	*β-actin*	F:	AGGGAAATCGTGCGTG
		R:	ATGATGCTGTTGTAGGTGGT
**12h PBS vs Poly(I:C) in liver**			
Nib0095610.1	*BAMBI*	F:	GCCTCATCCTTGTCCTGCTCATCA
		R:	CATCTGCTGCCGCTGGTCCTG
Nib0179480.1	*ULK2*	F:	CAGGTGGAACAGTTGGTGCTCTAC
		R:	GACGGCGGTGGAAGGATTGAG
Nib0206870.1	*CD118*	F:	AAGTTCGCAACAAGGACAGAG
		R:	CAGACCAGGACAGGATGAAGT
Nib0052830.1	*IL1B*	F:	TTGTGCCCTTGATGCCCAGAG
		R:	AACACAGTTCCTCCTCACATTG
**48h PBS vs Poly(I:C) in liver**			
Nib0080900.1	*TXNRD*	F:	CTGGTGGTTATTGGAGGTGGAT
		R:	CTGATGCTGATGCTGTTGATACTG
Nib0160990.1	*ABHD6*	F:	AGACTGCGGCGGTTATCG
		R:	CTGTGTTGGTGCGTGTTGTG
Nib0013800.1	*CYP24A1*	F:	AACATCCTCAGTGTGCGTCCT
		R:	CCAGTGCCTCGTGTTGTCTTG
Nib0166040.1	*IGFBP1*	F:	AGAGGCTGAGATGGAGAACACA
		R:	CTTCTTGCGGATGGCGATGAG
**12h PBS vs Poly(I:C) in Spleen**			
Nib0066250.1	*DDIT4*	F:	CAAACATGCCTGCCCTCTCTG
		R:	AGATCACTCCTGCTGCCATTGT
Nib0142290.1	*C3*	F:	TGTTGCGGTTGAAGTTGTGAATCC
		R:	CATTGGTCCTTGCGGTGATTGC
Nib0031000.1	*F8*	F:	AGCAGGCGAGGAGAAGGAGGAT
		R:	GATGAGCACGGCACCGATGAGT
Nib0184280.1	*CXCR2*	F:	CTGGACACCGTACCACATTGC
		R:	AACAGCCTCCTTCTGAACTTCTCT
**48h PBS vs Poly(I:C) in Spleen**			
Nib0106490.1	*ACTR2*	F:	TGAGACTGTGCGGATGTTGAAG
		R:	TGATGAGATGAGGCTGGAAGAGA
Nib0230860.1	*GABBR*	F:	CCAGGATTACCAGGACTCTTACC
		R:	GCAGCATCTTGTGAACTTCGT
Nib0091400.1	*CD38*	F:	CTGCTGTTGCTGCTGTAACGATT
		R:	AGGCTGACACAGGAACATCACA
Nib0000340.1	*SLC24A1*	F:	GTGCTGCTGTGCTTCGTCTAC
		R:	ACCTCCTCCTCCTTCTCTGTCT
